# Place preference induced by nucleus accumbens amphetamine is impaired by local blockade of Group II metabotropic glutamate receptors in rats

**DOI:** 10.1186/1471-2202-7-43

**Published:** 2006-05-30

**Authors:** Todor V Gerdjikov, Richard J Beninger

**Affiliations:** 1 Department of Psychology, Queen's University, Kingston, ON K7L 3N6, Canada; 2 Department of Psychiatry, Queen's University, Kingston, ON K7L 3N6, Canada

## Abstract

**Background:**

The nucleus accumbens (NAc) plays a critical role in amphetamine-produced conditioned place preference (CPP). In previous studies, NAc basal and amphetamine-produced DA transmission was altered by Group II mGluR agents. We tested whether NAc amphetamine CPP depends on Group II mGluR transmission.

**Results:**

NAc injections (0.5 μl/side) of the Group II mGluR antagonist (2 *S*)- *a*-ethylglutamic acid (EGLU: 0.01–0.8 μg but not 0.001 μg) impaired CPP. The drug did not block the acute locomotor effect of amphetamine.

**Conclusion:**

Results suggest that Group II mGluRs may be necessary for the establishment of NAc amphetamine-produced CPP. These receptors may also mediate other forms of reward-related learning dependent on this structure.

## Background

Metabotropic glutamate receptors (mGluRs) are implicated in learning and synaptic plasticity [1]. mGluRs have been classified into three groups: Group I, including mGluR1 and mGluR5; Group II, including mGluR2 and mGluR3; and Group III, including mGluR 4 and mGluR 6–8. Group I stimulates phospholipase C (PLC) and phosphoinositide hydrolysis, whereas Group II and Group III inhibit cyclic adenosine monophosphate (cAMP) formation most likely through a G_i_-type protein [2,3]. Both cAMP and PLC are widely implicated in synaptic plasticity [4]. Through their activity on G-proteins and other second messengers, mGluRs modulate ion channel conductances, transmission through ligand-gated channels, as well as the activation of immediate early genes. Therefore, mGluRs are well suited to provide a means through which glutamate can induce synaptic changes at the same synapses where it elicits fast responses. The role of Group I mGluRs in learning and plasticity has been characterized extensively. Group II mGluRs have received less attention [1].

There is evidence suggesting a role for Group II in synaptic plasticity in learning. Group II is involved in corticostriatal long term depression (LTD) in the nucleus accumbens (NAc) [5]. Behavioral work implicates Group II receptors in olfactory and fear learning [6,7] and in lever pressing for food [8,9]. The reported Group II mGluR modulation of reward-related learning is consistent with the role of these receptors in downregulating the cAMP/PKA cascade[10]. cAMP-dependent protein kinase (PKA) activation mediates the acquisition of learning [11] and of reward-related learning in particular [12].

Both reward-related learning and addiction to psychostimulants critically involve NAc dopamine (DA) and share many of the same intracellular signals [12-14]. Glutamate release is necessary for amphetamine- and cocaine-produced conditioned place preference (CPP) [15,16] and systemic mGluR antagonists impair cocaine self-administration in rats [17]. The role of Group II mGluRs in the acquisition of psychostimulant reward has not been addressed in pharmacological studies.

Group II mGluRs modulate DA transmission. Locally administered agonists reduce, whereas locally administered antagonists increase NAc DA levels [18,19]. Group II mGluR agonists also modulate amphetamine-produced DA release, enhancing it in drug-naïve baboons [20] and impairing it in amphetamine-sensitized rats [21]. In a recent study mGluR2 receptor knockout mice showed enhanced cocaine-produced CPP [22]. Results showing that Group II blockade enhanced basal DA release [19] suggest that mGluR2^-^^/^^- ^mutants may exhibit behaviors related to psychostimulant sensitization [23], explaining the hyperlocomotion in a novel environment and enhanced cocaine CPP observed in these mice. The acute role of Group II mGluRs in the acquisition of NAc psychostimulant-produced CPP has not been investigated.

In the present studies, we used CPP [24] to test the hypothesis that NAc DA-mediated learning depends on Group II mGluRs. A Group II mGluR antagonist was administered directly into NAc and the acquisition of CPP based on NAc amphetamine was assessed. We found that CPP was antagonized by the Group II antagonist. Part of this research has been presented in abstract form [25].

## Results

### Histology

**Figure 1:**
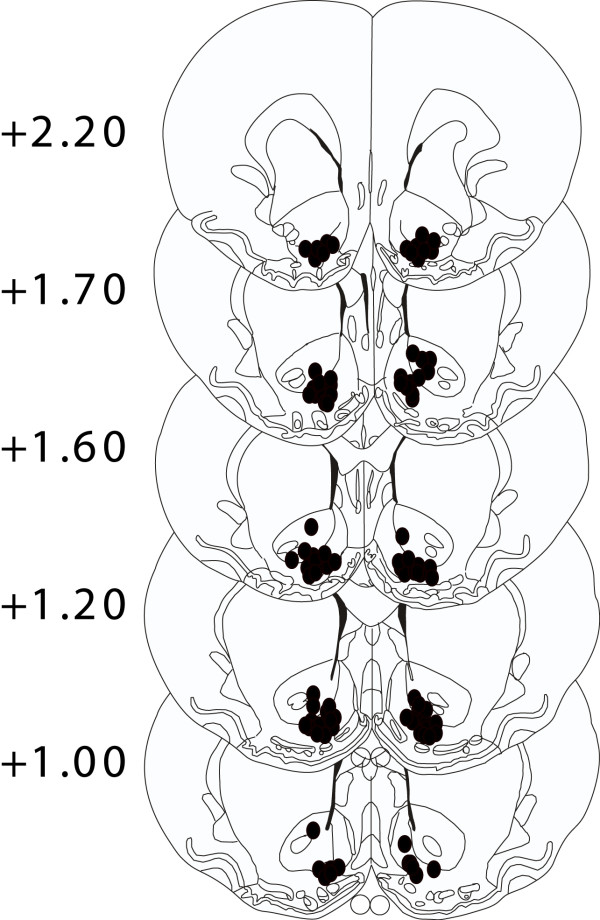
**Drawings of coronal sections through the nucleus accumbens indicating sites of infusion**. Injections of EGLU (0.0, 0.001, 0.01, 0.4 or 0.8 μg/0.5 μl/side) were followed by amphetamine injections (20 μg/0.5 μl/side) before conditioning sessions or 0.01 μg EGLU alone before conditioning sessions. Injector sites may appear fewer than the reported number of rats because of overlap of placements. Numbers to the left indicate distance (in millimeters) from bregma.

A total of 97 rats completed testing. Three rats failed to complete the study due to illness or technical problems. There was no relationship between the type and dose of drug and illness observed in these animals. Cannula placements were assessed for the remaining rats. A total of 24 rats was excluded leaving 73 rats for subsequent analyses. Figure 1 shows the location of cannula tips for all rats included in the analyses. Animals were classified as hits if the tips of both cannulae were located in the core or shell region of NAc.

### Time spent on each side during pre-exposure

The interpretation of CPP results is not straightforward if animals have a natural avoidance of the to-be-drug-paired side. In such a case, an apparent increase in time spent on that side after conditioning may be the result of decreased avoidance of the drug-paired side or simply habituation [24]. To check for bias, we averaged time spent on the side that would be paired with drug across the 3 habituation days and compared it to time spent on the side that would be paired with vehicle for each group. Paired samples t-tests revealed non-significant differences for all groups (see Table 1). Thus, rats did not avoid the to-be-drug-paired side during habituation and the CPP paradigm was unbiased.

### Tunnel time

A change in the time spent in the drug-paired side from habituation to test cannot be interpreted unambiguously as a change in place preference if time spent in the tunnel also changes. Thus, additional analyses were performed to compare tunnel time before and after conditioning (see Table 1). No significant differences were found.

### Place conditioning

**Figure 2:**
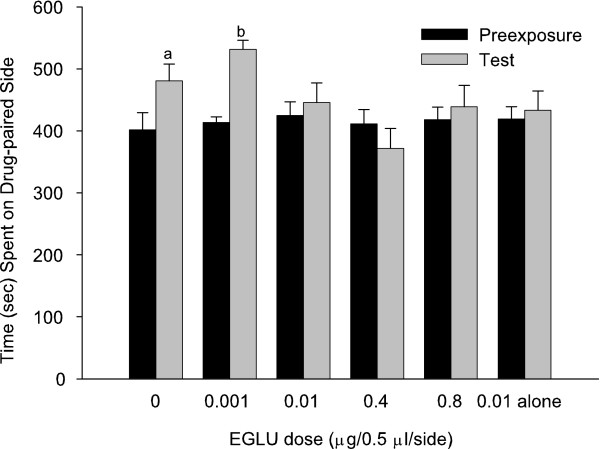
**Time (+ SEM) spent on the drug-paired side during the 15 min pre-exposure and the test sessions**. Testing occurred after conditioning with amphetamine (20 μg) and the Group II mGluR antagonist EGLU (0.0–0.8 μg) administered locally into NAc (bilaterally in 0.5 μl vehicle). One group received 0.01 μg EGLU alone, i.e. no amphetamine was administered before conditioning sessions. See Table 1 for number of animals per group. ^a^Significantly higher than time spent on drug-paired side on test day for rats receiving 0.4 μg of EGLU. ^b^Significantly higher than time spent on drug-paired side on test day for rats receiving 0.01–0.8 μg of EGLU.

To induce CPP NAc amphetamine was paired with one side of the apparatus over four conditioning days. EGLU was injected into NAc approximately ten minutes before amphetamine. Vehicle alone was paired with the other side of the apparatus on alternate days. CPP was analyzed using a dose (0.0, .001, .01, .4, .8 μg, or .01 alone) × phase (habituation vs. test) mixed ANOVA with phase as the repeated factor and time spent on the drug-paired side as the dependent variable. The ANOVA yielded a main effect of phase [*F*(1, 67) = 5.362, *p *< .05] and a dose × phase interaction [*F*(5, 67) = 2.77, *p *< .05]. A one-way simple effects between-subjects ANOVA testing the effect of dose during the habituation session was not significant. However, a one-way simple effects ANOVA testing the effect of dose during the test session produced a main effect of dose [*F*(5, 67) = 2.99, *p *< .05]. Pairwise comparisons revealed that on the test day rats conditioned with amphetamine alone spent more time on the drug-paired side than rats conditioned with amphetamine plus 0.4 μg of EGLU. Rats conditioned with amphetamine plus the lowest EGLU dose (0.001 μg) spent more time on the drug-paired side compared to all other groups except the group conditioned with amphetamine alone (Fisher LSD *posthoc *tests; all *p*s < .05). This confirmed the hypothesis that EGLU impairs CPP acquisition. Rats receiving 0.01 μg of EGLU but no amphetamine showed no significant preference or avoidance (Figure 2).

### Locomotor activity

**Figure 3:**
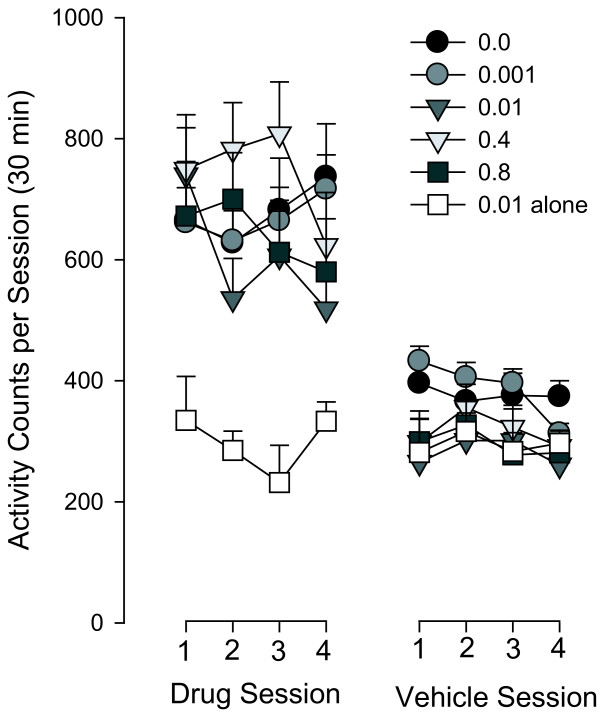
**Mean (+SEM) activity counts (per 30 min) during conditioning sessions**. Activity counts on the drug- (left panel) and vehicle-paired (right panel) sides are shown. Groups were treated with 0.0 – 0.8 μg EGLU followed by amphetamine (20 μg) immediately before placement on the corresponding side of the conditioning apparatus. One group received 0.1 μg EGLU alone, i.e. no amphetamine was administered before conditioning sessions. See Table 1 for number of animals per group.

Locomotor activity during conditioning sessions was analyzed for EGLU doses on the four amphetamine and four saline conditioning days yielding a dose (0.0, 0.001, 0.01, 0.4, 0.8 μg, 0.01 μg alone) × phase (amphetamine vs. saline) × day (4 days per phase) 3-way mixed ANOVA with phase and day as the within-subjects factors and total number of beam breaks per session as the dependent variable. The analysis revealed main effects of phase [*F*(1, 67) = 100.72, *p *< .001] and dose [*F*(5, 67) = 12.50, *p *< .001] and dose × phase [*F*(5, 67) = 5.47, *p *< .001] and dose × phase × day [*F*(15, 201) = 2.31, *p *< .01] interactions. In the drug phase, there was only a main effect of EGLU dose [*F*(5, 67) = 8.69, *p *< .001]. Animals receiving amphetamine or amphetamine plus EGLU (0.001, 0.01, 0.4 or 0.8 μg) showed higher locomotor activity than animals conditioned with 0.01 μg of EGLU alone (all *p*s < .05, Fisher LSD *posthoc *tests). In the saline phase, there was a main effect of EGLU dose [*F*(5, 67) = 5.35, *p *< .001]. The amphetamine group showed higher activity than the 0.01, 0.01 alone and 0.8 groups. The 0.001 μg group showed higher activity than the higher EGLU doses (0.01, 0.4, 0.8 and 0.01 alone; all *p*s < .05, Fisher LSD *posthoc *tests). Thus amphetamine increased locomotor activity both in groups that showed a CPP (0 and 0.001 doses) and in groups that did not (0.01, 0.4 and 0.8 doses). Although activity was significantly lower in all groups on vehicle days, the two groups that showed a place preference had higher locomotion in the drug-free phase (Figure 3).

## Discussion

In the present studies NAc amphetamine injections produced CPP. This effect was impaired by a Group II mGluR antagonist. The CPP paradigm was unbiased; rats did not show a systematic preference for one side of the apparatus over the other before conditioning. Also, the time rats spent in the tunnel before and after conditioning did not change significantly showing that increases in time spent on the drug-paired side were in fact due to changes in preference. If EGLU produced conditioned place avoidance on its own then simple additivity of this putative effect and the positive effect of amphetamine might explain the observed pattern of results. To test this, we injected one group of rats with EGLU alone on what would normally have been an amphetamine plus EGLU day using an EGLU dose that was found to affect amphetamine CPP (0.01 μg). On test day these rats did not show a preference or avoidance for the chamber normally paired with amphetamine. Thus putative place conditioning properties of EGLU do not appear to account for the observed results. Amphetamine stimulated locomotor activity. Activity among groups differed in drug-free vehicle sessions, with the groups that had received amphetamine alone or amphetamine plus 0.001 μg EGLU into NAc during conditioning being more active.

Groups that showed the CPP effect also showed higher activity in the vehicle phase. It is difficult to explain this observation. We know of no reports of a relationship between activity on *vehicle *conditioning days and the presence of a CPP effect. In our work we have repeatedly observed locomotor stimulation on *drug *conditioning days in groups that subsequently showed a CPP and groups that did not. Further studies are needed to assess the reliability of this finding and of activity differences among groups on vehicle conditioning days.

EGLU administration did not affect the acute increases in locomotor activity produced by amphetamine in the drug phase. EGLU has been reported to impair NAc amphetamine-produced locomotion using an amphetamine dose of 2.5 μg/0.5 μl/side [26]. We have previously reported that NAc amphetamine doses ranging from 5.0–20.0 μg produced similar levels of locomotor stimulation but a dose-dependent CPP effect (Beninger et al., 2003). For the present study we chose a dose of amphetamine (20 μg) that produced a significant CPP effect; this dose was eight times higher than the dose needed to produce motor stimulation in the study of Kim et al. (2000). The doses of EGLU (0.09 and 0.88 μg) that they used to reduce the locomotor stimulatory effect of amphetamine were comparable to the higher doses used here. Our failure to replicate their findings probably relates to our use of a substantially higher dose of amphetamine.

The NAc can be subdivided into core and shell subregions [27] and a number of studies suggest that these subregions may be differentially involved in reward-related learning and locomotion [28-32]. Our data did not permit a direct investigation of the relative contribution of the two subregions. An attempt was made to assess the possible role of the core and shell by separating the groups into core and shell placements and performing post hoc analyses. However only a few rats had both cannulae located within the core subregion. These rats were distributed unevenly among the groups making it impossible to statistically analyze the effect of region. It should be noted however that we have previously reported significant amphetamine CPP using the same vehicle volume and injection parameters with placements in both the core and shell [33]. In other work, lower injection volumes have been used to study the differential role of each subregion [34]. A more thorough investigation of the role of NAc subregions in CPP produced by local injections of amphetamine is awaited.

Dopaminergic innervation of the NAc, among other structures, has been widely implicated in natural appetitive behaviors and addiction [35]. NAc plays a critical role in the acquisition of amphetamine-produced CPP [36,37]. DA modulation of corticostriatal glutamatergic projections is believed to underlie reward-related learning [38-40]. mGluRs may be involved in this modulation. Group I blockade impaired corticostriatal synaptic plasticity [41]. Group I mGluRs have been implicated in the acquisition of drug reward [42,43] and in mediating DA agonist-produced changes in intracellular cascades widely implicated in memory and plasticity [1,44-46]. While Group II mGluRs have not been investigated as thoroughly in this context, several behavioral studies have demonstrated a role for Group II mGluRs in learning. Group II mGluR activation rescued a nonselective mGluR antagonist-produced impairment of the spontaneous improvement in lever-pressing for food in mice [9]. Group II activation also impaired lever-pressing for food in rats although this effect was schedule-dependent [8]. In a mouse study, systemic cocaine-produced CPP was enhanced in Group II mutants [22]. In contrast, we found that acute administrations of the Group II mGluR antagonist EGLU impairs the acquisition of NAc amphetamine CPP. Our results are not in disagreement with this study. The mutants showed a behavioral spectrum consistent with psychostimulant sensitization, including enhanced cocaine behavioral sensitization, hyperlocomotion in a novel environment and enhanced DA release after a systemic cocaine injection. This observation is supported by work showing that a locally administered mGluR II antagonist increased basal DA levels [19]. The observed phenotype may therefore reflect long term adaptations produced by changes in striatal DA rather than acute effects of Group II mGluR blockade.

Little is known about the mechanism though which Group II mGluRs modulate the acquisition of reward-related learning. Group II mGluR antagonist-produced impairments in the acquisition of amphetamine CPP may be mediated by effects on PKA. The acquisition of reward-related learning involves DA receptor-mediated PKA activation [12]. Both the inhibition and direct activation of PKA impair reward-related learning and learning in other tasks [6,33,47]. In an in vitro study, a group II antagonist enhanced the stimulation of cAMP formation by forskolin [48]. Both a Group II agonist and antagonist impaired learning in a step-down avoidance task [6]. The agonist-produced impairment was rescued by coinfusion of forskolin, suggesting that mGluR II may act in synchrony with other receptors including mGluR I and DA_1_-like receptors to produce an optimal level of PKA activation. In fact, work from our lab [33,49] has shown that activation of the cAMP/PKA cascade impairs NAc amphetamine-produced CPP – a finding that may parallel the effects of a mGluR antagonist on a tonically active Group II mGluR signal.

Group II mGluRs may modulate plasticity by altering NMDA-DA receptor interactions in NAc. NMDA receptors are necessary for DA-mediated reward-related learning [50]. Group II activation alters NMDA-produced intracellular signaling in striatal neurons [51]. Group II inhibition impairs NMDA-dependent hippocampal LTD [52] and corticostriatal NAc LTD [5]. NAc LTD may mediate NAc-dependent learning and psychostimulant-produced plasticity [53,54]. Reward-related learning is thought to be mediated by the selective strengthening of behaviorally relevant corticostriatal inputs to striatum [38-40]. Perhaps Group II mGluRs are involved in the selective depression of synapses not related to current input in effect adjusting the gain of behaviorally relevant glutamatergic efferents to the NAc [54]. Further work will have to test this intriguing possibility.

## Conclusion

We investigated the role of Group II mGluRs in reward-related learning using the CPP paradigm. This report is the first to test the effects of a Group II mGluR antagonist applied locally in the brain on the acquisition of CPP produced by NAc injections of amphetamine. We showed that the antagonist impaired NAc amphetamine-produced CPP. The results are consistent with previous research showing a role for mGluRs in memory and plasticity and DA-mediated reward-related learning. Group II mGluR modulation of DA effects in the NAc may involve changes in PKA activation, changes in NMDA signaling or both.

## Methods

### Animals and surgery

Male Wistar rats (Charles River, St. Constant, Quebec) weighing between 200–250 g on arrival were housed in pairs on a 12-hr reversed light-dark cycle (lights on at 19:00 hr) at an average temperature of 21°C, humidity 40–70%. Water and food (LabDiet 5001, PMI Nutrition Intl, Brentwood, MO) were freely available. Rats were handled for about 1 min on each of 5 consecutive days after arrival. The experimental protocol was approved by the Animal Care Committee at Queen's University. All animals were treated in full compliance with the Animals for Research Act and relevant guidelines set by the Canadian Council on Animal Care.

Approximately one wk after arrival at the colony, rats were anesthetized in an induction chamber using an inhalable anesthetic (5% isoflurane; Bimeda, Cambridge, ON) mixed with oxygen in a vaporizer system (Benson, Merkham, ON) and administered at 1.0 l/min. Anesthetized animals were fitted to a stereotaxic apparatus and isoflurane was administered at a concentration of 2% or as needed to maintain anesthesia. The head was adjusted so that lambda and bregma were on the same horizontal plane. For analgesia, buprenorphine hydrochloride in solution (0.15 mg/kg; Reckitt & Colman, Richmond, VA) was injected subcutaneously preoperatively. Ketoprofen (1.5 mg/kg; Merial, Baie d'Urfé, QC) was injected immediately after surgery and on three subsequent days postoperatively. The experimenter shaved the rat's head and applied betadine solution with a cotton tip applicator before incising the skin. Holes were drilled into the skull and 23 gauge (0.64 mm diameter) stainless-steel guide cannulae were chronically implanted bilaterally into the NAc, with coordinates: 1.6 mm anterior to bregma, 1.4 mm lateral to the midline and 6.7 mm ventral from the skull surface [55]. The guide cannulae were held in place by four stainless-steel screws and dental acrylic. Stainless steel wire stylets (0.31 mm diameter) flush with the end of the guide cannulae were put in place to prevent occlusion. Rats were allowed approximately one wk to recover before the start of behavioral testing. Behavioral data were analyzed if the tips of the cannulae were located in the core or shell region of NAc.

### Drug infusion

The Group II mGluR antagonist (2 *S*)- *a*-ethylglutamic acid (EGLU; Tocris, Ellisville, MO) was dissolved in saline before the beginning of the experiment and stored at -20°C. The pharmacological properties of this agent have been well documented. EGLU impaired presynaptic mGluR agonist-produced motoneuron depolarization believed to be mediated by Group II mGluRs and inhibited mGluR agonist binding in mGluR2-transfected cell membranes [56].

Amphetamine sulfate (USP, Rockvill, MD) was dissolved in saline daily before each set of injections. Central injections into the NAc were made with a microinfusion pump (KD Scientific, Holliston, MA). Injectors were glued to polyethylene tubing (0.75 mm o.d.) filled with distilled water. The tubing was connected to two 10 μl microsyringes (Microliter #701; Hamilton, Reno, NV) mounted on the microinfusion pump. Drugs were backloaded into the injectors by aspiration with the two syringes. Rats were hand-held as the experimenter removed the stylets from the guide cannulae and inserted the two injectors (0.31 mm o.d.). The injectors projected 1.2 mm beyond the guide cannulae.

EGLU (0.001, 0.01, 0.4 or 0.8 μg/0.5 μl/side) was injected bilaterally over 30 sec. The highest EGLU dose was similar to a dose previously reported to impair NAc amphetamine-produced locomotion [26]. After the drug was delivered, the injectors were left in place for another 30 sec to facilitate diffusion, after which they were slowly retracted from the guide cannulae. Amphetamine (20.0 μg/0.5 μl/side) was injected approximately 10 min later using the same procedure. This amphetamine dose was much higher than the one reported by Kim et al. (2000) and has been found to produce reliable CPP in previous studies from our lab (e.g. [49,57]). Place conditioning began immediately after amphetamine injection. Groups were tested drug-free.

### Apparatus

The 4 testing chambers each consisted of two rectangular compartments (38 × 27 × 34 cm) connected with a tunnel (8 × 8 × 8 cm). Two different spatial features were varied across the 4 testing chambers. The compartment walls were either urethane-sealed wood or alternating 1 cm-wide black and white vertical stripes and were covered with clear Plexiglas. The floor was either wire bars 1 cm apart running perpendicular to the tunnel, or a wire grid with openings of 1 cm^2^. This resulted in 4 possible compartment types, distributed as left or right compartment across 4 different testing chambers. Each compartment had a Plexiglas top with a number of circular ventilation holes. The tunnel was fitted with guillotine-type doors, which could be closed to prevent movement from one compartment to the other. Two infrared emitters and detectors (height 5.0 cm) in each compartment and 2 in the tunnel (height 5.0 cm) were used to monitor movement between and within compartments and to record time spent in each compartment and the tunnel. The number of beam breaks during conditioning sessions was used as a measure of locomotion. Each of the 4 testing chambers was housed in a dimly lit (7.5 Watt), sound-attenuated and ventilated wooden box. Indirect light reached the rat though the Plexiglas tops of the compartments. Data from the sensors were collected on a 6809 microcontroller using custom made software and transferred to a Macintosh computer for analyses. For further details of the apparatus see [58].

### Behavioral procedure

Training and testing occurred during the day (7:00–19:00 hr). Rats were tested in groups of 4 using a different testing chamber for each rat. The experimental protocol consisted of 3 habituation sessions, 8 conditioning sessions, and 1 test session. Each rat completed one session per day for a total of 12 days.

#### Habituation

At the start of each 15-min session the rat was placed in one compartment of the box – left compartment for half the rats and right compartment for the other half. Tunnel doors were open allowing the animals to move freely between the two compartments. Activity sensors recorded the amount of time spent on each side and in the tunnel.

#### Conditioning

Over 8 30-min sessions carried out on 8 consecutive days, mGluR antagonists and amphetamine were injected into the NAc on days 1, 3, 5 and 7, and vehicle on days 2, 4, 6 and 8. Injection of the mGluR antagonist was followed by an injection of amphetamine approximately 10 min later. Rats received only one injection on vehicle days. Immediately after amphetamine or vehicle injection, depending on conditioning day, the rat was placed in one of the two compartments with the tunnel doors closed preventing movement into the other compartment or the tunnel. Half the rats were confined to the left side on drug days and to the right side on vehicle days. The other half were confined to the right side on drug days and to the left side on vehicle days. In this way, any given compartment was paired with drug for some animals and with vehicle for others. Number of beam breaks was recorded for each rat to assess locomotion.

Six groups were tested. Five groups received amphetamine (20 μg/0.5 μl/side) immediately before placement into the conditioning compartment; these injections were preceded 10 min earlier by saline or EGLU doses of 0.001, 0.01, 0.4 or 0.8 μg/0.5 μl/side. One additional group received EGLU (0.01 μg) 10 min before and saline immediately before placement into the conditioning compartment. This group assessed possible effects of EGLU alone on side preference.

#### Testing

Testing occurred on the day immediately following conditioning. The session lasted 15 min and was identical to habituation sessions. The start side was the same for each rat on test and habituation days. Time spent on each side and in the tunnel was recorded.

### Data analysis

Paired samples *t*-tests were used to compare tunnel time before and after conditioning. To check for side bias, another set of *t*-tests compared time spent on the drug-paired side to time spent on the vehicle-paired side before conditioning. The amount of time spent on the drug-paired side was averaged across the 3 habituation days. CPP was analyzed using a mixed dose × phase (habituation vs. test) analysis of variance (ANOVA) followed by restricted ANOVA and pair-wise comparisons. In addition, the number of beam breaks for each rat on each of the 8 conditioning days was summed and used as an index of motor activity. Activity data were analyzed using a dose × session type × day mixed ANOVA.

### Histology

After completion of the experiment, rats were placed in an airtight chamber and euthanized with CO_2_. Brains were removed and preserved in a 10% formalin solution for at least 72 hrs. Coronal sections 60 μm thick from throughout the cannulated region were obtained by slicing the brains on a cryostat at -20°C. The sections were mounted on gelatin-coated glass slides and stained with cresyl violet. Judgments about the cannulae placements in the NAc were made by an observer who was blind to the results for individual animals. Brains with cannula tips in the shell and/or core regions of the NAc were included in subsequent analyses.

## Authors' contributions

TVG carried out the behavioral studies, participated in the design of the studies and the statistical analysis of the data, and drafted the manuscript. RJB conceived the study, participated in its design and coordination, and in revising the manuscript. Both authors read and approved the final manuscript.

## Figures and Tables

**Table 1:** Mean (± SEM) time (sec) spent on the drug-paired and vehicle-paired side before conditioning and in the tunnel before and after conditioning. The conditioned place preference paradigm was unbiased.

*Dose of EGLU/0.5 μl/side*	*N*	*Time in drug-paired side before conditioning*	*Time in veh-paired side before conditioning*	*Tunnel time before conditioning*	*Tunnel time after conditioning*
0.0 μg	9	401.6 (17.0)	446.1 (19.1)	52.3 (5.1)	48.8 (9.4)
0.001 μg	22	418.7 (19.9)	421.5 (20.1)	59.7 (3.7)	68.7 (6.5)
0.01 μg	12	431.4 (30.2)	413.6 (31.9)	58.6 (5.4)	55.1 (5.8)
0.4 μg	9	411.2 (22.0)	431.2 (24.4)	57.6 (4.7)	51.4 (6.4)
0.8 μg	9	418.2 (23.0)	439.1 (25.1)	48.0 (4.0)	52.3 (3.6)
0.01 μg alone	12	418.8 (20.4)	416.6 (21.1)	64.6 (6.0)	69.5 (8.1)

Abbreviations: Veh = vehicle.

*Note*. None of the pairs of means are significantly different.
